# Jarborandi, a New Medicine

**Published:** 1874-08

**Authors:** 


Jarborandi, a New Medicine.—A new medicine, with marvellous virtues, according to its sponsors, has been introduced and experimented with at the Hospital Baujon, Paris. An account of the action and characters of the medicine appears in the *Repertoire de Pharmacie* of March 25, from which we condense the following particulars. Dr. S. Continho, of Pernambuco, who claims to have discovered the properties of the plant, induced Professor Gubler to make a trial of it, and the account by that eminent physician corresponds exactly with the claims put forth by Dr. Continho.

The leaves and little twigs of the plant are broken up, and from four to six grams infused in a cupful of warm water. The infusion may be taken warm or cold; and in about ten minutes after administration the patient breaks out into a violent perspiration, which continues for four or five hours, and which is so thorough as to necessitate several changes of linen. At the same time a most abundant flow of saliva is promoted; so abundant, says M. Gubler, that speech is rendered almost impossible. He asserts that he has known patients eject *more than a litre* in less than two hours. Occasionally the medicine has induced diarrhœa. Its action is more rapid and more thorough if taken warm, and if the patient is well covered up in bed, but its effects are none the less certain under quite contrary conditions.

MM. Continho and Gubler justly assume that there is a great future for a drug of such capabilities as this *jarborandi* seems to possess. According to Prof. Baillon, the plant belongs to a species of the rue family, the *pilocarpus pinnatus: jarborandi,* it seems, is the Indian name for the plant. M. Continho slightly shakes our confidence in the miraculous power of his *protege* when he tells us that it is to be found in the interior of some of the northern provinces of Brazil; an expression which seems to bear a relationship to Dr. Bliss' famous cundurango formula, the herb which was only of value when procured "from the almost inaccessible slopes of the Andes." We shall hope for further enlightenment and evidence concerning this energetic diaphoretic.—*Chemist and Druggist.*

